# Molecularly defined extraintestinal pathogenic *Escherichia coli* status predicts virulence in a murine sepsis model better than does virotype, individual virulence genes, or clonal subset among *E. coli* ST131 isolates

**DOI:** 10.1080/21505594.2020.1747799

**Published:** 2020-04-07

**Authors:** Irene Merino, Stephen B. Porter, Brian Johnston, Connie Clabots, Paul Thuras, Patricia Ruiz-Garbajosa, Rafael Cantón, James R. Johnson

**Affiliations:** a Servicio de Microbiología, Hospital Universitario Ramón y Cajal and Instituto Ramón y Cajal de Investigación Sanitaria (IRYCIS), Madrid, Spain; b Spanish Network for Research in Infectious Diseases (REIPI), Madrid, Spain; c Infectious Diseases, Minneapolis Veterans Health Care System, Minneapolis, MN, USA; d Medicine, University of Minnesota, Minneapolis, MN, USA; e Mental Health Service Line, Minneapolis Veterans Health Care System, Minneapolis, MN, USA; f Psychiatry, University of Minnesota, Minneapolis, MN, USA

**Keywords:** *E. coli*, virulence, ExPEC, mouse sepsis model

## Abstract

Background: *Escherichia coli* ST131, mainly its *H*30 clade, is the leading cause of extraintestinal *E. coli* infections but its correlates of virulence are undefined. Materials and methods: We tested in a murine sepsis model 84 ST131 isolates that differed by country of origin (Spain vs. USA), clonal subset, resistance markers, and virulence genes (VGs). Virulence outcomes, including illness severity score (ISS) and “killer” status (>80% mouse lethality), were compared statistically with clonal subset, individual and combined VGs, molecularly defined extraintestinal and uropathogenic *E. coli* (ExPEC, UPEC) status, and country of origin. Results: Virulence varied widely by strain. Univariable correlates of median ISS and percent “killer” (outcomes if variable present vs. absent) included *pap* (ISS, 4.4 vs. 3.8; “killer”, 71% vs. 46%), *kpsMII* (4.1 vs. 2.3; 59% vs. 25%), K2/K100 (4.4 vs. 3.2; 77% vs. 41%), ExPEC (4.2 vs. 2.2; 62% vs. 17%), Spanish origin (4.3 vs. 3.1; 65% vs. 36%), and *H*30R1 subset (2.5 vs. 4.1; 35% vs. 59%). With multivariable adjustment, ExPEC status was the only consistently significantly predictive variable. Conclusion: Within ST131 the strongest predictor of experimental virulence was molecularly defined ExPEC status. Clonal subsets seemed to behave differently in the murine sepsis model by country of origin.

## Background

The pandemic extraintestinal pathogenic *Escherichia coli* (ExPEC) clone ST131 is a major contributor to the increasing incidence of extraintestinal *E. coli* infections, mainly bloodstream and urinary tract infections, especially those caused by fluoroquinolone-resistant or extended-spectrum beta-lactamase (ESBL)-producing strains. ST131 also occurs in the gut microbiota of healthy and institutionalized persons [1].

Recent studies have elucidated the complex population structure of ST131 and the corresponding lineage-specific strain characteristics [2–4]. These include divergent antimicrobial resistance profiles between the recently emerged pandemic clade *H*30 (clade C) (fluoroquinolone resistance-associated) and ancestral clades *H*41 (clade A) and *H*22 (clade B), and, within clade *H*30, between sister subsets *H*30R1 (subset C1; CTX-M-14 and CTX-M-27-associated) and *H*30Rx (subset C2; CTX-M-15-associated).

Like other *E. coli* clones from virulence-associated phylogroup B2, ST131 exhibits a broad range of genes that encode known or suspected virulence factors, hence are called virulence genes (VGs). Such VGs, which contribute to adherence, colonization, invasion, and/or persistence in the host, include siderophores (*iutA, fyuA, iroN*), adhesins (*fimH, pap, afa/dra, iha, yfcV*), toxins (*sat, vat*), protectins (*traT, iss*, capsule variants), and miscellaneous elements (*cvaC, ompT, usp, malX*). ST131’s VGs have been proposed as a possible reason for its dramatic dissemination and clinical emergence. Moreover, some lineages within ST131 have been associated with sepsis, worse clinical outcomes, and errors in empirical treatment [5,6]. Whether for ST131 particular VGs or combinations thereof are required for, or associated with, successful colonization, establishment of infection, or progression to severe disease is unclear.

Studies to date of the experimental virulence of ST131 in diverse animal hosts (mice, zebrafish, *Caenorhabditis elegans*, and *Galleria mellonella*) have yielded conflicting results [7–10]. Moreover, several authors have identified specific combinations of VGs, or “virotypes”, within ST131 [8,11,12] that in some studies predicted experimental virulence [8]. However, interpretation of these studies is impeded by their small sample size, inconsistent selection of VGs, limited attention to ST131 clonal subsets, and diversity of animal models, including some of the uncertain relevance to human infections.

Because of the importance of identifying potentially virulent ST131 strains, we sought here to identify among *E. coli* ST131 isolates associations of experimental virulence with diverse bacterial traits that could act as markers for said virulence, whether or not they directly determine it. For this, we used an established murine sepsis model and a comparatively large collection of well-characterized ST131 isolates of diverse ecological and geographical origins. We then compared experimental virulence results with the strains’ country of origin, virulence genotype, ST131 clonal subset, ESBL genotype, and fluoroquinolone resistance status.

## Methods

### Study collection and subtyping

A convenience sample of 84 diverse ST131 *E. coli* isolates from various previously published collections from our group was analyzed [9,13–17]. Geographical origin, year of testing, and ecological source are shown in Table 1. Whereas all isolates from Spain were tested in 2014, 81% of the isolates from USA were tested pre-2014. Because the control strains yielded consistent results across experiments and years (not shown), we assumed that any variation associated with the year of testing was due to geographical factors. Accordingly, to avoid possible bias, we also analyzed the variable “country”, despite its close correlation to “year of testing”.

**Table 1.** Characteristics of the 84 *Escherichia coli* ST131 studied isolates.

		No. of isolates (column %)			
Category	Specific characteristic	Total (*n* = 84)	Non-*H*30 (*n* = 24)	*H*30R1 (*n* = 23)	*H*30Rx (*n* = 37)
Year of testing	Pre2014	29 (34)	10 (42)	13 (56)	6 (16)
	2014	55 (66)	14 (58)	10 (44)	31 (84)
Country	USA	36 (43)	17 (71)	13 (56)	6 (16)
	Spain	48 (57)	7 (29)	10 (44)	31 (84)
Origin^a^	Blood	55 (66)	9 (38)	12 (52)	34 (92)
	Urine	20 (24)	10 (42)	8 (35)	2 (5)
	Fecal	4 (5)	4 (17)	0 (0)	0 (0)
	Other^b^	3 (4)	0 (0)	2 (9)	1 (3)
Resistance	FQ-R	60 (71)	3 (12)	21 (91)	36 (97)
	ESBL	27 (32)	1 (4)	0 (0)	26 (70)

FQ-R, fluoroquinolone-resistant; ESBL, extended-spectrum beta-lactamase.

^a^The origin of two isolates was not available.

^b^Origin of the “other” samples included wound, articular fluid, and kidney abscess drainage.

In previous studies, using PCR-based assays, the ST131 isolates were classified into established sublineages, including non-*H*30 (clades *H*41 and *H*22), *H*30 (clade C), and *H*30 subsets *H*30R1 and *H*30Rx (subclades C1 and C2) [2,18,19] (Table 1). ESBL genes were assessed by PCR and sequencing [20–22] and fluoroquinolone resistance by disk diffusion according to CLSI guidelines [23].

### Virulence genotype

**Figure 1.**
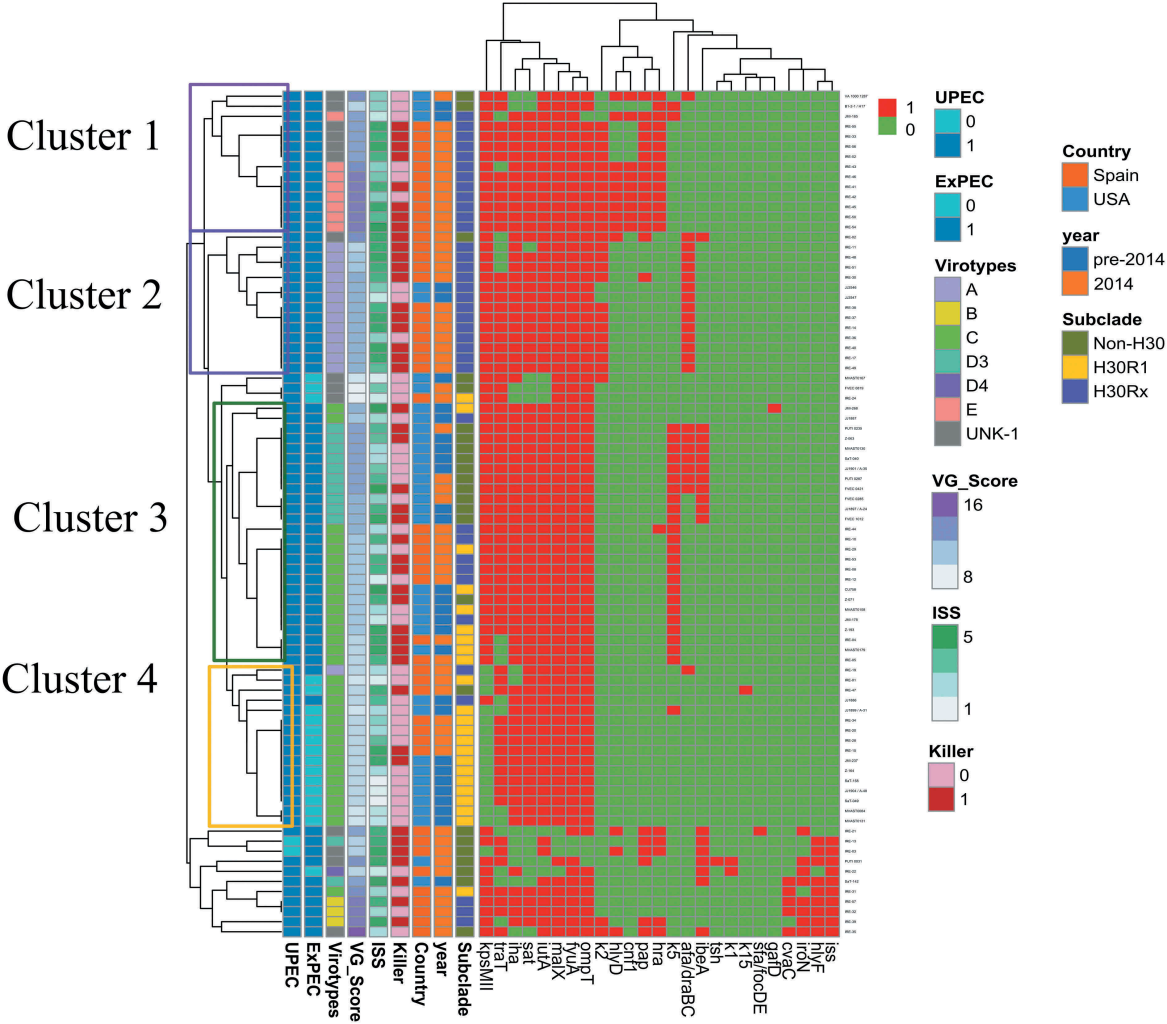
Heatmap of the aggregated virulence gene (VG) profiles for the 84 *Escherichia coli* ST131 study isolates. Only variably present VGs are shown. *pap*, presence of any of the *pap* operon genes tested; i.e., *papAH, papC, papEF, papG* (with alleles I, II, III). Red (1): gene present. Green (0): gene absent. ExPEC: extraintestinal pathogenic *E. coli*. UPEC: uropathogenic *E. coli*. UN: virotype undefined. ISS: illness severity score. This figure appears in color in the online version and black and white in the print version.

Forty-nine putative ExPEC-associated VGs and variants thereof (Table 2) were detected by an established multiplex PCR assay [9]. The VG score was calculated as the total number of VG operons detected. Based on established molecular definitions, isolates were classified as presumptive ExPEC if they carried ≥2 of *papAH* and/or *papC* (counted as one: P fimbriae), *sfa/focDE* (S and F1C fimbriae), *afa/dra* (Dr antigen-specific adhesin), *iutA* (aerobactin system), and *kpsMII* (group 2 capsules) [24], and as presumptive uropathogenic *E. coli* (UPEC) if they carried ≥3 of *chuA*, (heme uptake), *fyuA* (yersiniabactin system), *vat* (vacuolating autotransporter toxin), and *yfcV* (adhesin) [25]. These definitions did not necessarily correspond with the strain’s clinical source (i.e., extraintestinal clinical vs. fecal or intestinal source for ExPEC, and urinary vs non-urinary source for UPEC). VG profiles were classified into virotypes according to published criteria [8]. A heatmap showing VG presence/absence (Figure 1) was constructed using the pheatmap package within RStudio version 1.0.44 (RStudio Team 2016, RStudio: Integrated Development for R. RStudio, Inc., Boston, MA).

**Table 2.** Virulence gene (VG) distribution among clonal subsets detected in the 84 *Escherichia coli* ST131 studied isolates. Virulence genes^a,b.^

		No. of isolates (column %)				
Category	Specific gene	Total (*n* = 84)	Non-*H*30 (*n* = 24)	*H*30R1 (*n* = 23)	*H*30Rx (*n* = 37)	*p* value^c^
Adhesins	*pap^d^*	21 (25)	7 (29)	0 (0)	14 (38)	**<0.01**
	*papAH*	18 (21)	4 (17)	0 (0)	14 (38)	**<0. 01**
	*papC*	20 (24)	7 (29)	0 (0)	13 (35)	**<0. 01**
	*papEF*	20 (24)	7 (29)	0 (0)	13 (35)	**<0. 01**
	*papG^e^*	17 (20)	4 (17)	0 (0)	13 (35)	**<0.01**
	*papGII*	16 (19)	3 (12)	0 (0)	13 (35)	**<0.01**
	*papGIII*	1 (1)	1 (4)	0 (0)	0 (0)	0.56
	*sfa/focDE*	1 (1)	1 (4)	0 (0)	0 (0)	0.56
	*focG*	1 (1)	1 (4)	0 (0)	0 (0)	0.56
	*afa/dra*	23 (27)	9 (38)	0 (0)	14 (38)	**<0.01**
	*iha*	72 (86)	15 (62)	21 (91)	36 (97)	**<0.01**
	*gafD*	1 (1)	0 (0)	1 (4)	0 (0)	0.27
	*hra*	20 (24)	6 (25)	0 (0)	14 (38)	**<0.01**
Toxins	*hlyD*	13 (16)	5 (21)	0 (0)	8 (22)	0.03
	*hlyF*	9 (11)	5 (21)	1 (4)	3 (8)	0.22
	*cnf1*	9 (11)	1 (4)	0 (0)	8 (22)	**0.01**
	*cdtB*	1 (1)	1 (4)	0 (0)	0 (0)	0.56
	*sat*	71 (84)	13 (54)	22 (96)	36 (97)	**<0.01**
	*tsh*	1 (1)	1 (4)	0 (0)	0 (0)	0.56
Siderophores	*iroN*	8 (10)	5 (21)	0 (0)	3 (8)	0.04
	*fyuA*	82 (98)	22 (92)	23 (100)	37 (100)	0.15
	*ireA*	1 (1)	1 (4)	0 (0)	0 (0)	0.56
	*iutA*	78 (93)	19 (79)	22 (96)	37 (100)	**<0.01**
Protectins	*kpsMII*	68 (81)	22 (92)	10 (44)	36 (97)	**<0.01**
	K1	2 (29)	2 (8)	0 (0)	0 (0)	0.15
	K5	27 (32)	12 (50)	8 (35)	7 (19)	0.04
	K15	1 (1)	1 (4)	0 (0)	0 (0)	0.56
	K2/K100	26 (31)	3 (12)	0 (0)	23 (88)	**<0.01**
	*iss*	10 (12)	6 (25)	1 (4)	3 (8)	0.11
	*traT*	67 (80)	20 (83)	17 (74)	30 (81)	0.74
Miscellaneous	*ibeA*	18 (21)	18 (75)	0 (0)	0 (0)	**<0.01**
	*ompT*	80 (95)	21 (88)	23 (100)	36 (97)	0.20
	*cvaC*	5 (6)	2 (8)	1 (4)	2 (5)	0.86
	*malX*	80 (95)	20 (83)	23 (100)	37 (100)	**0.01**

^a^*pap*, pilus associated with pyelonephritis; *sfa/focDE,* S and F1C fimbriae; *focG,* pilus tip of F1C fimbrie; *afa/dra,* Dr antigen-specific adhesin; *iha,* non-hemagglutinin adhesin; *gafD,* N-acetyl-D-glucosamine-specific fimbriae adhesin; *hra,* heat-resistant agglutinin; *hlyD,* alpha-hemolysin; *cnf1,* cytotoxic necrotizing factor 1; *cdtB,* cytolethal distending toxin; *sat,* secreted autotransporter toxin; *tsh,* temperature-sensitive hemagglutinin; *iroN,* catecholate siderophore receptor; *fyuA,* ferric yersiniabactin uptake receptor; *ireA,* siderophore receptor; *iutA,* ferric aerobactin receptor; *kpsMII,* group-2 capsule synthesis; K1, K5, K15, K2/K100, group-2 capsule variants; *iss* and *traT,* outer membrane proteins involved in serum survival; *ibeA,* invasion of brain endothelium; *ompT,* outer membrane protease T; *cvaC,* microcin V; *malX,* pathogenicity-associated island marker.

^b^*usp* (bacteriocin), *yfcV* (fimbriae) and *fimH* (type 1 fimbriae) were found in all isolates. *sfa* (S fimbriae), *afaE8* (afimbrial adhesin), *bmaE* (adhesin), f17 (F17 c fimbriae), *clpG* (CS31A adhesin), *pic* (serin protease), *vat* (vacuolating autotransporter), *east1* (heat-stable toxin), *kps*III (group-3 capsule synthesis), *rfc* (LPS synthesis), H7, *clbB* (peptide-polyketide synthase), and *clbN* (non-ribosomal synthetase) were not detected in any isolate.

^c^Chi-square and Fisher’s exact tests were used for *p* values. After Bonferroni correction, only those *p* values <.02 were considered significant. Statistically significant values are in bold.

^d^*pap* was considered positive if *papAH, papC, papEF*, or *papG* were positive.

^e^*papG* was considered positive if allele II or III of *papG* was positive. No isolate was positive for allele I of *papG.*

### Experimental virulence

In vivo virulence was assessed previously using a well-established murine subcutaneous sepsis model [13,26] at the Minneapolis Veteran Affairs Medical Center (MN, USA) according to animal use protocol 120,603, as approved by the local Institutional Animal Care and Use Committee. The sepsis model results for the present isolates were reported elsewhere [13,14].

For this model, female pathogen-free outbred Swiss-Webster mice were inoculated subcutaneously with approximately 10^9^ CFU/mL log-phase bacteria in 0.2 mL saline, as described previously [13,26]. Mouse health was assessed twice daily for 3 days post-challenge by experienced researchers unaware of strain identity, following a strict protocol and using positive control strain CFT073 (high lethality) and negative control strain MG1655 (zero illness or lethality). Mice were classified daily as to maximal illness severity, which ranged from 1 (healthy) to 5 (dead), with intermediate scores 2 (barely ill), 3 (moderately ill), and 4 (severely ill). Results for the controls were consistent during all the experiments, regardless of year (data not shown).

The variables used as metrics of the study isolates’ virulence potential included overall mean illness severity score (ISS), a continuous variable obtained by averaging the daily illness severity scores for the mice challenged with a given isolate, and “killer” status, defined based on death of ≥80% of the challenged mice [15]. Each test strain was assessed initially in five mice, followed by another five mice if the initial testing did not yield a consistent result (i.e., lethality or survival for four or five of the initially challenged mice). To minimize the risk of a possible cohort bias, mice from a given shipment were assigned to different test strains using a formal randomization scheme.

### Statistical analysis

Continuous variables were described as means and standard deviations (SDs) or medians and interquartile ranges (IQRs) and were compared using a t-test and ANOVA or the Mann–Whitney and Kruskal–Wallis tests, as appropriate. Dichotomous variables were described using frequencies and percentages and were compared using a chi-square test or Fisher’s exact test, as appropriate. The criterion for statistical significance was p < 0.05, with Bonferroni correction for multiple comparisons. To avoid possible bias involving the variables “year of testing” and “country of origin”, analyses were repeated after stratification by year and country.

Univariable and multivariable regression analysis (simple regression, for ISS; logistic regression, for “killer” status) were used to assess the predictive power of independent variables with and without adjustment for collinearity between them. For multivariable analysis, only those bacterial traits were included that in univariable analyses predicted one or both of the experimental virulence outcomes, whether overall or after stratification by year or country. For use in multivariable modeling, the qualifying univariable predictors were divided into a core set (ExPEC status, belonging to the *H*30R1 clonal subset, and year of testing) and a supplementary set (VG score, genes *pap, kpsMII*, K2/K100).

Two methods were used for variable entry into the multivariable models, i.e., forced and stepwise. The forced-entry method was applied first to only the core set of candidate predictor variables, then to the core plus supplementary variable sets combined. The stepwise method was applied to both variable sets combined. Data were analyzed using STATA (Stata Statistical Software: Release 11. College Station, TX: StataCorp LP).

## Results

### Subtyping, resistance traits, and VG-derived classifications

The 84 study isolates were diverse for clonal background, VG content, country of origin, year of testing, and resistance markers (Table 1). Overall, of the studied clonal variants, the *H*30 clade was most common (71%, 60/84), and was divided between the *H*30Rx subset (62%, 37/60) and the *H*30R1 subset (38%, 23/60).

Of the 49 studied VGs and variants, 35 were detected in at least one isolate each. VGs were distributed significantly by clonal subset (Table 2). However, with stratification by year of testing and country of origin, the only statistically significant differences involved *kpsMII* and *ibeA* (Supplementary material tables S1-S4). The mean VG score was 11.8 (SD 1.9) overall but was lower among *H*30R1 isolates (mean 10.2, SD 1.2) than among *H*30Rx (mean 12.6, SD 1.7) and non-*H*30 isolates (mean 12.1, SD 1.9) (p < 0.01, *H*30R1 vs. *H*30Rx or non-*H*30). Even with stratification by country of origin and year of testing, these differences remained statistically significant. By contrast, within a given clonal subset, VG scores did not differ significantly by year of testing or country of origin (Supplementary material Table S5).

Overall, regardless of source of isolation, 79% (66/84) of isolates qualified molecularly as ExPEC and 98% (82/84) as UPEC. Fewer *H*30R1 isolates than others qualified as ExPEC, both overall (39% vs 93%, p < 0.01) (Table 3) and with stratification by year of testing and country of origin (Supplementary material table S6). ExPEC status was associated with higher VG scores, both overall (ExPEC, median 12 [IQR 2], vs. non-ExPEC, 10 [IQR 1]: p < 0.01) and with stratification by year of testing and country of origin (Supplementary material Table S7).

**Table 3.** Virulence gene combinations according to clonal subset classification among 84 *Escherichia coli* ST131 studied isolates.

		No. of isolates (column %)			
Category		Total (*n* = 84)	Non-*H*30 (*n* = 24)	*H*30R1 (*n* = 23)	*H*30Rx (*n* = 37)
ExPEC		66 (79)	20 (83)	9 (39)	37 (100)
UPEC		82 (98)	22 (92)	23 (100)	37 (100)
Virotype	A	14 (17)	0 (0)	0 (0)	14 (38)
	B	3 (4)	0 (0)	0 (0)	3 (8)
	C	32 (38)	2 (8)	22 (96)	8 (22)
	D	13 (15)	13 (54)	0 (0)	0 (0)
	E	8 (9)	0 (0)	0 (0)	8 (22)
	UN	14 (17)	9 (38)	1 (4)	4 (11)

ExPEC, extraintestinal pathogenic *E. coli*. UPEC, uropathogenic *E. coli*. UN, undefined virotype.

Overall, 83% (70/84) of isolates fulfilled criteria for a described virotype, most commonly C (38%, 32/84), followed by A (17%, 14/84) and D (15%, 13/84). Virotypes A, B, D and E were found only in certain clonal subsets, while virotype C was found in all three subsets (Table 3).

The 35 detected VGs occurred in 38 distinct combinations (38 VG profiles; Figure 1). Whereas most profiles (66%, 25/38) occurred in a single isolate each, the two most common profiles were repeated 9 and 10 times each. The heatmap based on VG content showed four main clusters (Cluster1-4) of related VG profiles, which corresponded roughly with virotypes and clonal subsets. Cluster 1 and 2 grouped mainly *H*30Rx isolates and corresponded mostly with virotype E and A, respectively. Cluster 3 split into two main subclusters; one grouped all non-*H*30 isolates (all virotype D3), whereas the other grouped a mix of isolates from different clonal backgrounds (virotype C). Finally, Cluster 4 grouped mainly *H*30R1 isolates and corresponded mostly with virotype C. These four clusters differed mostly for presence/absence of *pap, kpsMII*, specific group 2 capsular variants, *hra, afa/dra, ibeA*, and *traT* (Figure 1).

### Experimental virulence outcomes vs. bacterial characteristics

In the murine sepsis model, ISS was fairly high overall (median 3.9, on a 1–5 scale), but varied greatly by isolate (IQR 2.2), with approximately half (54%, 44/84) of isolates qualifying as “killers”. ISS and “killer” status were significantly associated (median ISS: “killers”, 4.7 [IQR 0.5], vs. other isolates, 2.4 [IQR 1.1], p < 0.001). ISS was higher for those isolates tested in 2014 (median ISS: 4.3 [IQR 2.5] vs 2.5 [IQR 1.8], p = 0.03) or from Spain (median ISS: 4.3 [IQR 1.9] vs. 3.1 [2.4], p = 0.08). Likewise, the “killer” fraction was higher for isolates tested in 2014 (62% vs. 35%, p = 0.02) or from Spain (65% vs. 36%, p = 0.01) (Supplementary table S8).

### Virulence vs. subclades and resistance markers (split by year/country)

Compared with other isolates, *H*30R1 isolates had a significantly lower “killer” fraction (35% vs. 59%, p = 0.047) and somewhat lower ISS values (median ISS: *H*30R1 isolates, 2.5 [IQR 2.8], vs. non-*H*30R1 isolates, 4.1 [IQR 1.9], p = 0.07) (Table 4). However, stratification by year of testing and country eliminated *H*30R1’s (negative) association with “killer” status, and showed that its association with lower ISS values was limited to isolates tested in 2014 or from Spain (in either subset, median ISS: *H*30R1 isolates, 2.8 [IQR 1.9], vs. non-*H*30R1 isolates, 4.3 [IQR 1.5]: p = 0.04). By contrast, *H*30Rx isolates tested in 2014 or from Spain showed significantly higher ISS and were more often “killer” than *H*30Rx isolates tested pre-2014 or from USA (Supplementary material Table S8). Neither ESBL production nor FQ resistance was associated significantly with ISS or “killer” status.

**Table 4.** Virulence outcomes according to bacterial characteristics among 84 *Escherichia coli* ST131 studied isolates.

No. of isolates with bacterial characteristic		ISS (median, IQR)	*p* value^a^	Killer status (row %)	*p* value^a^
Subsets	Non-*H*30 (*n* = 24)	3.9 (1.8)	0.20	13 (54)	0.12
	*H*30R1 (*n* = 23)	2.5 (2.8)^b^		8 (35)^a^	
	*H*30Rx (*n* = 37)	4.3 (1.1)		23 (62)	
ESBL	Yes (*n* = 27)	4.3 (1.7)	0.20	17 (63)	0.18
	No (*n* = 57)	3.8 (2.4)		27 (47)	
FQ	R (*n* = 60)	3.9 (2.3)	0.57	31 (52)	0.84
	S (*n* = 24)	3.9 (1.9)		13 (54)	
Virotype	A (*n* = 14)	4.4 (1.5)	0.37	10 (71)	0.49
	B (*n* = 3)	4.3 (2.2)		2 (67)	
	C (*n* = 32)	2.8 (2.2)		13 (41)	
	D3 (*n* = 12)	3.9 (1.6)		7 (58)	
	D4 (*n* = 1)	1.7 (0)		0 (0)	
	E (*n* = 8)	3.5 (1.6)		4 (50)	
	UN (*n* = 14)	4.3 (1.9)		8 (57)	
ExPEC	Yes (*n* = 66)	4.2 (1.9)	**<0.001**	41 (62)	**0.001**
	No (*n* = 18)	2.2 (1.9)		3 (17)	
UPEC	Yes (*n* = 82)	3.9 (2.2)	0.15	42 (51)	0.50
	No (*n* = 2)	4.6 (0.3)		2 (100)	

ISS, illness severity score. ESBL, extended-spectrum beta-lactamases. FQ, fluoroquinolone. R, resistant. S, susceptible. UN, undefined virotype. ExPEC, extraintestinal pathogenic *E. coli*. UPEC, uropathogenic *E. coli.*

^a^Chi-square and Fisher’s exact tests (qualitative dependent variable) and Mann–Whitney and Kruskal–Wallis tests (quantitative dependent variable) were used for p values. Statistically significant values are in bold.

^b^For two-group comparison of *H*30R1 vs. others: for ISS, *p* = 0.07; for killer status, *p* = 0.047.

### Virulence vs. individual VGs (split by year/country)

Overall, of the 49 studied individual VGs, *pap, kpsMII*, and K2/K100 were associated significantly with ISS and “killer” status; the median ISS and percent “killer” were significantly higher for isolates with vs. without the particular gene (Table 5). However, with stratification by year or country, many of these comparisons lost statistical significance or differed inconsistently by subset (not shown).

**Table 5.** Results for the univariable and multivariable analysis of statistically significant predictors of experimental virulence for the 84 *Escherichia coli* ST131 studied isolates.

	Illness severity score (ISS)						“Killer” status					
	Univariable		Multivariable^a^ (forced-entry method)		Multivariable (stepwise method)		Univariable		Multivariable^b^ (forced-entry method)		Multivariable(stepwise method)	
Predictor^c^	*β* coef.	*P* value^d^	*β* coef.	*P* value^d^	*β* coef.	*P* value^d^	OR (CI)	*P* value^d^	OR (CI)	*P* value^d^	OR (CI)	*P* value^d^
*pap*	0.68	**0.03**	0.16	0.67	0.05	0.68	2.9 (1.0–8.5)	**0.049**	1.4 (0.3–6.9)	0.65	2.9 (1.0–8.5)	**0.049**
*kpsMII*	0.98	**0.003**	−0.32	0.52	−0.10	0.50	4.3 (1.2–14.7)	**0.02**	0.5 (0.05–6.1)	0.62	4.3 (1.2–14.7)	**0.02**
K2	0.82	**0.003**	0.47	0.15	0.13	0.24	4.7 (1.6–13.5)	**0.004**	3.7 (0.9–15.5)	0.07	3.4 (1.1–10.0)	**0.03**
VG score	0.18	**0.01**	−0.10	0.31	−0.08	0.54	1.2 (1.0–1.5)	0.07	0.7 (0.5–1.1)	0.15	1.2 (1.0–1.5)	0.07
ExPEC	1.33	**<0.001**	1.72	**0.001**	1.22	**<0.001**	8.2 (2.2–31.2)	**0.002**	23.2 (1.9–278.7)	**0.01**	5.98 (1.5–23.5)	**0.01**
*H*30R1	−0.70	**0.02**	0.25	0.48	0.08	0.50	0.37 (0.1–1.0)	**0.047**	1.5 (0.4–6.6)	0.56	0.37 (0.1–1.0)	0.05
Year (2014)^e^	0.75	**0.006**	0.47	0.09	0.57	**0.02**	3.1 (1.2–7.9)	**0.02**	2.0 (0.6–6.3)	0.24	3.1 (1.2–7.9)	**0.02**

*β* coef, Beta coefficient. OR (CI), odds ratio (95% confidence interval). ExPEC: Extraintestinal pathogenic *E. coli.*

^a^Multivariable linear regression: for final model with all variables included, *r*^2^ = 0.29 (*p* < 0.001).

^b^Multivariable logistic regression: for final model with all variables included, *r*^2^ = 0.23 (*p* = 0.002).

^c^Variables shown are the significant univariable predictors of one or both virulence outcomes.

^d^Statistically significant *P* values are in bold.

^e^Year of testing correlated roughly with country of origin (rho = 0.84, *p* < 0.001), so can be considered a surrogate for that trait.

### Virulence vs. ExPEC/UPEC status, VG score, virotypes, and VG profiles (in relation to year and country)

We analyzed virulence in relation to four types of combinations of VGs: molecularly defined ExPEC/UPEC status, VG score, virotype, and VG profile. Overall, ExPEC status (79% prevalence) was significantly associated with both ISS (median ISS: ExPEC, 4.2 [IQR 1.9], vs. non-ExPEC, 2.2 [IQR 1.9], p < 0.001) and “killer” status (ExPEC, 62%, vs. non-ExPEC, 17%: p = 0.001). After stratification by year of testing and country of origin, ExPEC status maintained its association with ISS, although the “killer” fraction was significantly higher only among ExPEC isolates from Spain or tested in 2014 (Supplementary Table S9). By contrast, UPEC status was unsuitable for statistical analysis due to its 98% overall prevalence.

Overall, VG score was correlated only weakly with ISS (rho = 0.29, p = 0.008), and was slightly higher among “killer” isolates (median score: 12 [“killers”], vs. 11 [others], p = 0.03). With stratification by year of testing, VG score was not associated with either virulence endpoint in either subgroup. With stratification by country, the correlation of VG score with ISS was only marginally statistically significant among isolates from the USA (rho = 0.37, p = 0.03) and was not significant among isolates from Spain (rho = 0.18, p = 0.23). By contrast, VG score was not associated with “killer” status in either subgroup (data not shown).

Virotype was not associated with experimental virulence (Table 4). Due to already-small group sizes, these analyses were nor stratified by year and country. Finally, aggregate VG profiles (n = 38) grouped isolates with very different experimental virulence (Figure 1), without statistically significant virulence differences between profiles (p = 0.16). Additionally, the exploration of diverse combinations of VGs, as selected based on inspection of the heatmap, identified none other than ExPEC status that significantly predicted experimental virulence (not shown).

### Multivariable analysis

Given the multiple significant univariable predictors of virulence, and these variables’ associations with one another, multivariable analysis (with both forced entry and forward stepwise entry) was used to clarify primary associations and to allow adjustment for year of testing, which served as a proxy for the country of origin. Candidate predictor variables included a core set (*H*30R1, ExPEC status, year 2014) and a supplemental set based on VGs (*pap, kpsMII*, K*2*, VG score).

For predicting ISS, the initial forced-entry multiple regression model, with candidate predictors ExPEC, *H*30R1, and year 2014, identified as significant predictors both ExPEC (beta 1.4, p < 0.001) and year 2014 (beta 0.61, p = 0.02); *H*30R1 lost significance (beta 0.22, p = 0.50). The extended forced-entry model, which included the four supplemental variables as additional candidate predictors, identified ExPEC as the only significant multivariable predictor (beta 1.72, p = 0.001). The stepwise model, in which all seven variables were included as candidate predictors, yielded substantially similar results: the only significant predictors retained in the final model were ExPEC status (beta 1.2, p < 0.001) and, with lower predictive power and marginal statistical significance, year 2014 (beta 0.57, p = 0.02).

Similarly, for predicting “killer” status, the initial forced-entry logistic regression model, with candidate predictors ExPEC status, *H*30R1, and year 2014, identified as significant predictors both ExPEC (OR 9,3, CI 1,8–47,6) and year 2014 (OR 2,9, CI 1,0–8,1); *H*30R1 lost significance (OR 1,4, CI 0.4–5.7). The extended forced-entry model, which included the four supplemental variables as additional candidate predictors, identified ExPEC as the only significant multivariable predictor of “killer” status (OR 8.2, CI 2.2–31.2). The stepwise model yielded substantially similar results: the only significant multivariable predictors retained in the final model were ExPEC (OR 6.0, CI 1.5–23.5) and, with lower predictive power and marginal statistical significance, K2 (OR 3.5, 1.1–10.0) (Table 5).

## Discussion

In this study we analyzed a large collection of well-characterized *E. coli* ST131 isolates for associations between experimental virulence, as assessed in a murine sepsis model, and diverse bacterial traits, including clonal subsets, resistance markers, and virulence genotype. For this, we analyzed virulence genotype in multiple ways, including as both individual VGs and various combinations of VGs, i.e., molecular ExPEC and UPEC status, virotype, aggregate VG profile, and VG score.

Despite all strains being ST131, their experimental virulence in the murine sepsis model varied widely, both overall and within most subsets of the population, as defined based on diverse bacterial characteristics (e.g., clonal subsets, virotypes or VG score). This provided an opportunity to search for bacterial traits that correspond with experimental virulence.

According to the univariable analyses, four types of bacterial traits (i.e., specific clonal backgrounds, individual VGs, and VG combinations, and country of origin/tested in 2014) were significantly associated with ISS and “killer” status. To summarize: first, of the studied clonal subsets, *H*30R1 was associated negatively with both virulence endpoints, whereas a subset of *H*30Rx isolates (those from Spain or tested in 2014) was associated positively with these endpoints. Second, among the 49 studied VGs, *pap, kpsMII and* K2/K100 were associated positively with one or both virulence endpoints, although these associations varied by year and/or country. Third, of the studied VG combinations, only molecularly defined ExPEC status (robustly) and VG score (marginally) were associated (both, positively) with the virulence endpoints. Lastly, experimental virulence also varied overall in relation to year of testing (a surrogate for country of origin), probably due in part to country-specific differences in clonal subset distribution. Indeed, with stratification by clonal subset, only *H*30Rx isolates exhibited this by-year (i.e., by-country) difference in experimental virulence.

Previously reported results for a subset of the present isolates showed highly variable experimental virulence, with a trend toward lower virulence for *H*30R1 isolates [13,14]. Other studies of experimental virulence and ST131 likewise have yielded inconsistent results, not only across different animal models (mice, zebrafish, *C. elegans*, and *G. mellonella*) but even within a given model. For example, although initial results using the murine sepsis model suggested that ST131 was highly virulent [27], subsequent studies showed marked virulence variability [13,14,28]. Our results confirm this overall variability, notwithstanding a relatively high average virulence level. Additionally, with our comparatively large strain set, we were able to document in an univariable analysis significantly lower virulence for *H*30R1 isolates and, among isolates from Spain, higher virulence for *H*30Rx isolates.

Notably, certain individual VGs (*pap, kpsMII*, and K2/K100) were associated with both virulence and the *H*30Rx subset (vs. the *H*30R1 subset), especially among isolates from Spain. This is consistent with – and may partially explain – the greater observed virulence of Spanish *H*30Rx isolates, especially because virulence associations were stronger for VGs than for clonal subsets. Indeed, previous studies support a possible direct virulence contribution from the K2 capsule in non-ST131 clonal backgrounds [12,29,30]. These findings suggest that VG content and/or combinations of VGs (i.e., ExPEC molecular definition and VG score) could predict, and may determine, experimental virulence.

By contrast with the univariable analysis, the results of the multivariable analysis showed molecular ExPEC status as the strongest and only consistently significant predictor of experimental virulence, followed distantly by year of testing (which is a surrogate for the country of origin) and K2/K100. This finding may explain the initial observation of differences in experimental virulence across clonal subsets, which also differ for their molecularly defined ExPEC fraction (i.e., higher in *H*30Rx and lower in *H*30R1).

With the multivariable adjustment, whereas the individual VGs that were univariable predictors of experimental virulence lost statistical significance, ExPEC status remained a significant predictor. This may be explained by ExPEC status accounting for the influence of the individual VGs on virulence outcomes because the molecular definition of ExPEC includes those genes [14]. The very wide confidence interval around the OR for ExPEC suggests a need for studies involving more isolates, ideally of different countries of origin to test geographical impact.

By contrast with molecularly defined ExPEC status, neither virotype nor aggregate VG profiles significantly predicted virulence, which conflicts with previous findings [8]. Such inconsistencies across studies indicate that the described ST131 virotypes or even more extensive VGs profiles (as shown here), although associated with clonal background, [12,31,32] are insufficient to reliably predict experimental virulence. Although some of our negative findings may reflect in part the small numbers per subgroup after stratification by virotype, extended VG profiles, year of testing, and/or country of origin, even our overall analysis failed to replicate certain associations noted in previous studies, despite our greater total number of isolates.

Our study has some limitations. First, the murine sepsis model mimics only partially the pathogenesis of sepsis in humans, despite being standard in the field; outbred mice may vary in their response to infection, possibly contributing to experimental variability, although also presumably improving generalizability; and the use of only female mice conceivably could bias the results.

Second, our isolates were tested at different times, although temporal effects were addressed analytically by stratification by year of testing, and seem unlikely given the temporal stability of results for the control strains and for clonal subsets other than *H*30Rx. Third, our virulence genotyping relied on DNA detection for a limited set of VGs. Conceivably, expression/regulation of these or other (unaddressed) VGs linked to these may underlie the observed associations between VG presence and experimental virulence, and/or account for the residual unexplained virulence variation.

Fourth, the molecular definitions used for ExPEC and UPEC refer to the presence/absence of specific VGs, so do not track reliably with the source of isolation. However, they predict biological ExPEC status, defined as a strain’s intrinsic ability to cause extraintestinal infection, more accurately than does clinical source or presentation. Fifth, due to VG genotype variability within *E. coli*, strains that qualify as molecular UPEC do not necessarily qualify as ExPEC and vice versa, regardless of source; here, UPEC status was too prevalent for valid statistical analysis. Sixth, for some comparisons, statistical power was reduced by stratification by country and year of testing, and by the low prevalence of certain variables.

The study has also notable strengths. These include the large number of strains tested (making it, to our knowledge, the largest study to date of experimental virulence of ST131 strains in any animal model); the use of an established sepsis model that among those available most closely mimics human disease; attention to an extensive range of bacterial traits, including single and combined VGs and different ST131 subsets; and use of multiple statistical approaches, including multivariable modeling.

In conclusion, we found considerable variability in experimental virulence between and within the different ST131 clonal subsets, which differed significantly for VG content, ExPEC status, and virotype. With the multivariable adjustment, ExPEC status was the only consistently significant outcome predictor. Thus, composite markers such as ExPEC status are useful for identifying potentially virulent ST131 strains. These findings may help in devising screening tests and identifying targets for therapeutic or preventive interventions against infections caused by ST131 strains. They also indicate a need to study further the virulence determinants of ST131 (including possibly with *in vitro* assays such as serum resistance or survival in phagocytes), and to identify an explanation other than sepsis-causing ability for ST131’s impressive epidemiologic success.

## Supplementary material

The supplementary data for this article can be accessed here.
