# Supplementary material for: Molecularly defined extraintestinal pathogenic Escherichia coli status predicts virulence in a murine sepsis model better than does virotype, individual virulence genes, or clonal subset among E. coli ST131 isolates
Source: Virulence. 2020 Apr 7;11(1):327–36. doi: 10.1080/21505594.2020.1747799 (PMC7161687; doi:10.1080/21505594.2020.1747799)
Supplement: Supplemental Material [file kvir-11-01-1747799-s001.docx]

Table S1. Virulence gene (VG) distribution among clonal subsets detected in the 48 Spanish *Escherichia coli* ST131 studied isolates.

| Virulence genes^a,b^ | | No. of isolates (column %) | | | |  |
| --- | --- | --- | --- | --- | --- | --- |
| Category | Specific gene | Total  (n = 48) | Non-*H*30  (n = 7) | *H*30R1  (n = 10) | *H*30Rx  (n = 31) | p value^c^ |
| Adhesins | *pap^d^* | 18 (38) | 5 (71) | 0 (0) | 13 (42) | **0.003** |
|  | *papAH* | 16 (33) | 3 (43) | 0 (0) | 13 (42) | 0.03 |
|  | *papC* | 17 (35) | 5 (71) | 0 (0) | 12 (39) | **0.004** |
|  | *papEF* | 17 (35) | 5 (71) | 0 (0) | 12 (39) | **0.004** |
|  | *papG^e^* | 14 (29) | 2 (29) | 0 (0) | 12 (39) | 0.05 |
|  | *papGII* | 13 (27) | 1 (14) | 0 (0) | 12 (39) | 0.03 |
|  | *papGIII* | 1 (2) | 1 (14) | 0 (0) | 0 | 0.15 |
|  | *sfa/focDE* | 1 (2) | 1 (14) | 0 (0) | 0 (0) | 0.15 |
|  | *afa/dra* | 13 (27) | 1 (14) | 0 (0) | 12 (39) | 0.03 |
|  | *iha* | 41 (85) | 3 (43) | 8 (80) | 30 (97) | **0.001** |
|  | *gafD* | 0 (0) | 0 (0) | 0 (0) | 0 (0) | NA |
|  | *hra* | 17 (35) | 4 (57) | 0 (0) | 13 (42) | 0.02 |
| Toxins | *hlyD* | 11 (23) | 4 (57) | 0 (0) | 7 (23) | 0.02 |
|  | *hlyF* | 7 (15) | 3 (43) | 1 (10) | 3 (10) | 0.1 |
|  | *cnf1* | 7 (15) | 0 (0) | 0 (0) | 7 (23) | 0.13 |
|  | *cdtB* | 1 (2) | 1 (14) | 0 (0) | 0 (0) | 0.15 |
|  | *sat* | 41 (85) | 2 (29) | 9 (90) | 30 (97) | **< 0.001** |
|  | *tsh* | 0 (0) | 0 (0) | 0 (0) | 0 (0) | NA |
| Siderophores | *iroN* | 6 (12) | 3 (43) | 0 (0) | 3 (10) | 0.04 |
|  | *fyuA* | 46 (96) | 5 (71) | 10 (100) | 31 (100) | 0.02 |
|  | *ireA* | 0 (0) | 0 (0) | 0 (0) | 0 (0) | NA |
|  | *iutA* | 45 (94) | 5 (71) | 9 (90) | 31 (100) | 0.02 |
| Protectins | *kpsMII* | 40 (83) | 5 (71) | 5 (50) | 30 (97) | **0.002** |
|  | K1 | 1 (2) | 1 (14) | 0 (0) | 0 (0) | 0.15 |
|  | K5 | 8 (17) | 0 (0) | 3 (30) | 5 (16) | 0.32 |
|  | K15 | 1 (2) | 1 (14) | 0 (0) | 0 (0) | 0.15 |
|  | K2/K100 | 25 (52) | 2 (29) | 0 (0) | 23 (74) | **< 0.001** |
|  | *iss* | 8 (17) | 4 (57) | 1 (10) | 3 (10) | 0.02 |
|  | *traT* | 38 (79) | 4 (57) | 8 (80) | 26 (84) | 0.32 |
| Miscellaneous | *ibeA* | 6 (12) | 6 (86) | 0 (0) | 0 (0) | **< 0.001** |
|  | *ompT* | 46 (96) | 5 (71) | 10 (100) | 31 (100) | 0.02 |
|  | *cvaC* | 4 (8) | 1 (14) | 1 (10) | 2 (6) | 0.77 |
|  | *malX* | 44 (92) | 3 (43) | 10 (100) | 31 (100) | **< 0.001** |

^a^*pap*: pilus associated with pyelonephritis; *sfa/focDE*: S and F1C fimbriae; *afa/dra*: Dr antigen-specific adhesin; *iha*: non-hemagglutinin adhesin; *gafD*: N-acetyl-D-glucosamine-specific fimbriae adhesin; *hra*: heat-resistant agglutinin; *hlyD*: alpha-hemolysin; *cnf1*: cytotoxic necrotizing factor 1; *cdtB*: cytolethal distending toxin; *sat*: secreted autotransporter toxin; *tsh*: temperature-sensitive hemagglutinin; *iroN*: catecholate siderophore receptor; *fyuA*: ferric yersiniabactin uptake receptor; *ireA*: siderophore receptor; *iutA*: ferric aerobactin receptor; *kpsMII*: group-2 capsule synthesis; K1, K5, K15, K2/K100: group-2 capsule variants; *iss* and *traT*: outer membrane proteins involved in serum survival; *ibeA*: invasion of brain endothelium; *ompT*: outer membrane protease T; *cvaC*: microcin V; *malX*: pathogenicity-associated island marker.

^b^*usp* (bacteriocin), *yfcV* (fimbriae) and *fimH* (type 1 fimbriae) were found in all isolates. *sfa* (S fimbriae), *afaE8* (afimbrial adhesin), *bmaE* (adhesin), f17 (F17c fimbriae), *clpG* (CS31A adhesin), *pic* (serin protease), *vat* (vacuolating autotransporter), *east1* (heat-stable toxin), *kps*III (group-3 capsule synthesis), *rfc* (LPS synthesis), H7, *clbB* (peptide-polyketide synthase), and *clbN* (non-ribosomal synthetase) were not detected in any isolate.

^c^Chi-square and Fisher's exact tests were used for p values. After Bonferroni correction, only those p values < 0.008 were considered significant. Statistically significant values are in bold.

*^d^pap* was considered positive if *papAH, papC, papEF*, or *papG* were positive.

*^e^papG* was considered positive if allele II or III of *papG* was positive. No isolate was positive for allele I of *papG.*

Table S2. Virulence gene (VG) distribution among clonal subsets detected in the 36 *Escherichia coli* ST131 studied isolates from the USA

| Virulence genes^a,b^ | | No. of isolates (column %) | | | |  |
| --- | --- | --- | --- | --- | --- | --- |
| Category | Specific gene | Total  (n = 36) | Non-*H*30  (n = 17) | *H*30R1  (n = 13) | *H*30Rx  (n = 6) | p value^c^ |
| Adhesins | *pap^d^* | 3 (8) | 2 (12) | 0 (0) | 1 (17) | 0.38 |
|  | *papAH* | 2 (6) | 1 (6) | 0 (0) | 1 (17) | 0.43 |
|  | *papC* | 3 (8) | 2 (12) | 0 (0) | 1 (17) | 0.38 |
|  | *papEF* | 3 (8) | 2 (12) | 0 (0) | 1 (17) | 0.38 |
|  | *papG^e^* | 3 (8) | 2 (12) | 0 (0) | 1 (17) | 0.38 |
|  | *papGII* | 3 (8) | 2 (12) | 0 (0) | 1 (17) | 0.38 |
|  | *papGIII* | 0 (0) | 0 (0) | 0 (0) | 0 (0) | NA |
|  | *sfa/focDE* | 0 (0) | 0 (0) | 0 (0) | 0 (0) | NA |
|  | *afa/dra* | 10 (28) | 8 (47) | 0 (0) | 2 (33) | 0.01 |
|  | *iha* | 31 (86) | 12 (71) | 13 (100) | 6 (100) | 0.07 |
|  | *gafD* | 1 (3) | 0 (0) | 1 (8) | 0 (0) | 0.53 |
|  | *hra* | 3 (8) | 2 (12) | 0 (0) | 1 (17) | 0.38 |
| Toxins | *hlyD* | 2 (6) | 1 (6) | 0 (0) | 1 (17) | 0.43 |
|  | *hlyF* | 2 (6) | 2 (12) | 0 (0) | 0 (0) | 0.65 |
|  | *cnf1* | 2 (6) | 1 (6) | 0 (0) | 1 (17) | 0.43 |
|  | *cdtB* | 0 (0) | 0 (0) | 0 (0) | 0 (0) | NA |
|  | *sat* | 30 (83) | 11 (65) | 13 (100) | 6 (100) | 0.02 |
|  | *tsh* | 1 (3) | 1 (6) | 0 (0) | 0 (0) | 1.0 |
| Siderophores | *iroN* | 2 (6) | 2 (12) | 0 (0) | 0 (0) | 0.65 |
|  | *fyuA* | 36 (100) | 17 (100) | 13 (100) | 6 (100) | NA |
|  | *ireA* | 1 (3) | 1 (6) | 0 (0) | 0 (0) | 1.0 |
|  | *iutA* | 33 (92) | 14 (82) | 13 (100) | 6 (100) | 0.27 |
| Protectins | *kpsMII* | 28 (78) | 17 (100) | 5 (38) | 6 (100) | **< 0.001** |
|  | K1 | 1 (3) | 1 (6) | 0 (0) | 0 (0) | 1.0 |
|  | K5 | 19 (53) | 12 (71) | 5 (38) | 2 (33) | 0.13 |
|  | K15 | 0 (0) | 0 (0) | 0 (0) | 0 (0) | NA |
|  | K2/K100 | 1 (3) | 1 (6) | 0 (0) | 0 (0) | 1.0 |
|  | *iss* | 2 (6) | 2 (12) | 0 (0) | 0 (0) | 0.65 |
|  | *traT* | 29 (81) | 16 (94) | 9 (69) | 4 (67) | 0.16 |
| Miscellaneous | *ibeA* | 12 (33) | 12 (71) | 0 (0) | 0 (0) | **< 0.001** |
|  | *ompT* | 34 (94) | 16 (94) | 13 (100) | 5 (83) | 0.43 |
|  | *cvaC* | 1 (3) | 1 (6) | 0 (0) | 0 (0) | 1.0 |
|  | *malX* | 36 (100) | 17 (100) | 13 (100) | 6 (100) | NA |

^a^*pap*: pilus associated with pyelonephritis; *sfa/focDE*: S and F1C fimbriae; *afa/dra*: Dr antigen-specific adhesin; *iha*: non-hemagglutinin adhesin; *gafD*: N-acetyl-D-glucosamine-specific fimbriae adhesin; *hra*: heat-resistant agglutinin; *hlyD*: alpha-hemolysin; *cnf1*: cytotoxic necrotizing factor 1; *cdtB*: cytolethal distending toxin; *sat*: secreted autotransporter toxin; *tsh*: temperature-sensitive hemagglutinin; *iroN*: catecholate siderophore receptor; *fyuA*: ferric yersiniabactin uptake receptor; *ireA*: siderophore receptor; *iutA*: ferric aerobactin receptor; *kpsMII*: group-2 capsule synthesis; K1, K5, K15, K2/K100: group-2 capsule variants; *iss* and *traT*: outer membrane proteins involved in serum survival; *ibeA*: invasion of brain endothelium; *ompT*: outer membrane protease T; *cvaC*: microcin V; *malX*: pathogenicity-associated island marker.

^b^*usp* (bacteriocin), *yfcV* (fimbriae) and *fimH* (type 1 fimbriae) were found in all isolates. *sfa* (S fimbriae), *afaE8* (afimbrial adhesin), *bmaE* (adhesin), f17 (F17c fimbriae), *clpG* (CS31A adhesin), *pic* (serin protease), *vat* (vacuolating autotransporter), *east1* (heat-stable toxin), *kps*III (group-3 capsule synthesis), *rfc* (LPS synthesis), H7, *clbB* (peptide-polyketide synthase), and *clbN* (non-ribosomal synthetase) were not detected in any isolate.

^c^Chi-square and Fisher's exact tests were used for p values. After Bonferroni correction, only those p values < 0.008 were considered significant. Statistically significant values are in bold.

*^d^pap* was considered positive if *papAH, papC, papEF*, or *papG* were positive.

*^e^papG* was considered positive if allele II or III of *papG* was positive. No isolate was positive for allele I of *papG.*

Table S3. Virulence gene (VG) distribution among clonal subsets detected in the *Escherichia coli* ST131 studied isolates tested prior to 2014.

| Virulence genes^a,b^ | | No. of isolates tested pre-2014 (column %) | | | |  |
| --- | --- | --- | --- | --- | --- | --- |
| Category | Specific gene | Total  (n = 29) | Non-*H*30  (n = 10) | *H*30R1  (n = 13) | *H*30Rx  (n = 6) | p value^c^ |
| Adhesins | *pap^d^* | 1 (3) | 0 (0) | 0 (0) | 1 (17) | 0.21 |
|  | *papAH* | 1 (3) | 0 (0) | 0 (0) | 1 (17) | 0.21 |
|  | *papC* | 1 (3) | 0 (0) | 0 (0) | 1 (17) | 0.21 |
|  | *papEF* | 1 (3) | 0 (0) | 0 (0) | 1 (17) | 0.21 |
|  | *papG^e^* | 1 (3) | 0 (0) | 0 (0) | 1 (17) | 0.21 |
|  | *papGII* | 1 (3) | 0 (0) | 0 (0) | 1 (17) | 0.21 |
|  | *papGIII* | 0 (0) | 0 (0) | 0 (0) | 0 (0) | NA |
|  | *sfa/focDE* | 0 (0) | 0 (0) | 0 (0) | 0 (0) | NA |
|  | *afa/dra* | 6 (21) | 4 (40) | 0 (0) | 2 (33) | 0.03 |
|  | *iha* | 27 (93) | 8 (80) | 13 (100) | 6 (100) | 0.15 |
|  | *gafD* | 1 (3) | 0 (0) | 1 (8) | 0 (0) | 1.0 |
|  | *hra* | 2 (7) | 1 (10) | 0 (0) | 1 (17) | 0.30 |
| Toxins | *hlyD* | 1 (3) | 0 (0) | 0 (0) | 1 (17) | 0.21 |
|  | *hlyF* | 1 (3) | 1 (10) | 0 (0) | 0 (0) | 0.55 |
|  | *cnf1* | 1 (3) | 0 (0) | 0 (0) | 1 (17) | 0.21 |
|  | *cdtB* | 0 (0) | 0 (0) | 0 (0) | 0 (0) | NA |
|  | *sat* | 26 (90) | 7 (70) | 13 (100) | 6 (100) | 0.04 |
|  | *tsh* | 0 (0) | 0 (0) | 0 (0) | 0 (0) | NA |
| Siderophores | *iroN* | 1 (3) | 1 (10) | 0 (0) | 0 (0) | 0.55 |
|  | *fyuA* | 29 (100) | 10 (100) | 13 (100) | 6 (100) | NA |
|  | *ireA* | 0 (0) | 0 (0) | 0 (0) | 0 (0) | NA |
|  | *iutA* | 28 (97) | 9 (90) | 13 (100) | 6 (100) | 0.55 |
| Protectins | *kpsMII* | 21 (72) | 10 (100) | 5 (38) | 6 (100) | **0.001** |
|  | K1 | 0 (0) | 0 (0) | 0 (0) | 0 (0) | NA |
|  | K5 | 15 (52) | 8 (80) | 5 (38) | 2 (33) | 0.08 |
|  | K15 | 0 (0) | 0 (0) | 0 (0) | 0 (0) | NA |
|  | K2/K100 | 1 (3) | 1 (10) | 0 (0) | 0 (0) | 0.55 |
|  | *iss* | 1 (3) | 1 (10) | 0 (0) | 0 (0) | 0.55 |
|  | *traT* | 22 (76) | 9 (90) | 9 (69) | 4 (67) | 0.53 |
| Miscellaneous | *ibeA* | 7 (24) | 7 (70) | 0 (0) | 0 (0) | **< 0.001** |
|  | *ompT* | 28 (97) | 10 (100) | 13 (100) | 5 (83) | 0.21 |
|  | *cvaC* | 1 (3) | 1 (10) | 0 (0) | 0 (0) | 0.55 |
|  | *malX* | 29 (100) | 10 (100) | 13 (100) | 6 (100) | NA |

^a^*pap*: pilus associated with pyelonephritis; *sfa/focDE*: S and F1C fimbriae; *afa/dra*: Dr antigen-specific adhesin; *iha*: non-hemagglutinin adhesin; *gafD*: N-acetyl-D-glucosamine-specific fimbriae adhesin; *hra*: heat-resistant agglutinin; *hlyD*: alpha-hemolysin; *cnf1*: cytotoxic necrotizing factor 1; *cdtB*: cytolethal distending toxin; *sat*: secreted autotransporter toxin; *tsh*: temperature-sensitive hemagglutinin; *iroN*: catecholate siderophore receptor; *fyuA*: ferric yersiniabactin uptake receptor; *ireA*: siderophore receptor; *iutA*: ferric aerobactin receptor; *kpsMII*: group-2 capsule synthesis; K1, K5, K15, K2/K100: group-2 capsule variants; *iss* and *traT*: outer membrane proteins involved in serum survival; *ibeA*: invasion of brain endothelium; *ompT*: outer membrane protease T; *cvaC*: microcin V; *malX*: pathogenicity-associated island marker.

^b^*usp* (bacteriocin), *yfcV* (fimbriae) and *fimH* (type 1 fimbriae) were found in all isolates. *sfa* (S fimbriae), *afaE8* (afimbrial adhesin), *bmaE* (adhesin), f17 (F17c fimbriae), *clpG* (CS31A adhesin), *pic* (serin protease), *vat* (vacuolating autotransporter), *east1* (heat-stable toxin), *kps*III (group-3 capsule synthesis), *rfc* (LPS synthesis), H7, *clbB* (peptide-polyketide synthase), and *clbN* (non-ribosomal synthetase) were not detected in any isolate.

^c^Chi-square and Fisher's exact tests were used for p values. After Bonferroni correction, only those p values < .008 were considered significant. Statistically significant values are in bold.

*^d^pap* was considered positive if *papAH, papC, papEF*, or *papG* were positive.

*^e^papG* was considered positive if allele II or III of *papG* was positive. No isolate was positive for allele I of *papG.*

Table S4. Virulence gene (VG) distribution among clonal subsets detected in the *Escherichia coli* ST131 studied isolates tested in 2014.

| Virulence genes^a,b^ | | No. of isolates tested in 2014 (column %) | | | |  |
| --- | --- | --- | --- | --- | --- | --- |
| Category | Specific gene | Total  (n = 55) | Non-*H*30  (n = 14) | *H*30R1  (n = 10) | *H*30Rx  (n = 31) | p value^c^ |
| Adhesins | *pap^d^* | 20 (36) | 7 (50) | 0 (0) | 13 (42) | 0.02 |
|  | *papAH* | 17 (31) | 4 (29) | 0 (0) | 13 (42) | 0.03 |
|  | *papC* | 19 (35) | 7 (50) | 0 (0) | 12 (39) | 0.02 |
|  | *papEF* | 19 (35) | 7 (50) | 0 (0) | 12 (39) | 0.02 |
|  | *papG^e^* | 16 (29) | 4 (29) | 0 (0) | 12 (39) | 0.05 |
|  | *papGII* | 15 (27) | 3 (21) | 0 (0) | 12 (39) | 0.04 |
|  | *papGIII* | 1 (2) | 1 (7) | 0 (0) | 0 (0) | 0.44 |
|  | *sfa/focDE* | 1 (2) | 1 (7) | 0 (0) | 0 (0) | 0.44 |
|  | *afa/dra* | 17 (31) | 5 (36) | 0 (0) | 12 (39) | 0.05 |
|  | *iha* | 45 (82) | 7 (50) | 8 (80) | 30 (97) | **< 0.001** |
|  | *gafD* | 0 (0) | 0 (0) | 0 (0) | 0 (0) | NA |
|  | *hra* | 18 (33) | 5 (36) | 0 (0) | 13 (42) | 0.03 |
| Toxins | *hlyD* | 12 (22) | 5 (36) | 0 (0) | 7 (23) | 0.11 |
|  | *hlyF* | 8 (15) | 4 (29) | 1 (10) | 3 (10) | 0.25 |
|  | *cnf1* | 8 (15) | 1 (7) | 0 (0) | 7 (23) | 0.21 |
|  | *cdtB* | 1 (2) | 1 (7) | 0 (0) | 0 (0) | 0.44 |
|  | *sat* | 45 (82) | 6 (43) | 9 (90) | 30 (97) | **< 0.001** |
|  | *tsh* | 1 (2) | 1 (7) | 0 (0) | 0 (0) | 0.44 |
| Siderophores | *iroN* | (13) | 4 (29) | 0 (0) | 3 (10) | 0.09 |
|  | *fyuA* | 53 (96) | 12 (86) | 10 (100) | 31 (100) | 0.09 |
|  | *ireA* | 1 (2) | 1 (7) | 0 (0) | 0 (0) | 0.44 |
|  | *iutA* | 50 (91) | 10 (71) | 9 (90) | 31 (100) | **0.006** |
| Protectins | *kpsMII* | 47 (86) | 12 (86) | 5 (50) | 30 (97) | **0.001** |
|  | K1 | 2 (4) | 2 (14) | 0 (0) | 0 (0) | 0.09 |
|  | K5 | 12 (22) | 4 (29) | 3 (30) | 5 (16) | 0.49 |
|  | K15 | 1 (2) | 1 (7) | 0 (0) | 0 (0) | 0.44 |
|  | K2/K100 | 25 (45) | 2 (14) | 0 (0) | 23 (74) | **< 0.001** |
|  | *iss* | 9 (16) | 5 (36) | 1 (10) | 3 (10) | 0.11 |
|  | *traT* | 45 (82) | 11 (79) | 8 (80) | 26 (84) | 0.90 |
| Miscellaneous | *ibeA* | 11 (20) | 11 (79) | 0 (0) | 0 (0) | **< 0.001** |
|  | *ompT* | 52 (95) | 11 (79) | 10 (100) | 31 (100) | 0.02 |
|  | *cvaC* | 4 (7) | 1 (7) | 1 (10) | 2 (6) | 1.0 |
|  | *malX* | 52 (93) | 10 (71) | 10 (100) | 31 (100) | **0.004** |

^a^*pap*: pilus associated with pyelonephritis; *sfa/focDE*: S and F1C fimbriae; *afa/dra*: Dr antigen-specific adhesin; *iha*: non-hemagglutinin adhesin; *gafD*: N-acetyl-D-glucosamine-specific fimbriae adhesin; *hra*: heat-resistant agglutinin; *hlyD*: alpha-hemolysin; *cnf1*: cytotoxic necrotizing factor 1; *cdtB*: cytolethal distending toxin; *sat*: secreted autotransporter toxin; *tsh*: temperature-sensitive hemagglutinin; *iroN*: catecholate siderophore receptor; *fyuA*: ferric yersiniabactin uptake receptor; *ireA*: siderophore receptor; *iutA*: ferric aerobactin receptor; *kpsMII*: group-2 capsule synthesis; K1, K5, K15, K2/K100: group-2 capsule variants; *iss* and *traT*: outer membrane proteins involved in serum survival; *ibeA*: invasion of brain endothelium; *ompT*: outer membrane protease T; *cvaC*: microcin V; *malX*: pathogenicity-associated island marker.

^b^*usp* (bacteriocin), *yfcV* (fimbriae) and *fimH* (type 1 fimbriae) were found in all isolates. *sfa* (S fimbriae), *afaE8* (afimbrial adhesin), *bmaE* (adhesin), f17 (F17c fimbriae), *clpG* (CS31A adhesin), *pic* (serin protease), *vat* (vacuolating autotransporter), *east1* (heat-stable toxin), *kps*III (group-3 capsule synthesis), *rfc* (LPS synthesis), H7, *clbB* (peptide-polyketide synthase), and *clbN* (non-ribosomal synthetase) were not detected in any isolate.

^c^Chi-square and Fisher's exact tests were used for p values. After Bonferroni correction, only those p values < .008 were considered significant. Statistically significant values are in bold.

*^d^pap* was considered positive if *papAH, papC, papEF*, or *papG* were positive.

*^e^papG* was considered positive if allele II or III of *papG* was positive. No isolate was positive for allele I of *papG.*

Table S5. Virulence score of *Escherichia coli* ST131 studied isolates tested according to clonal subset, year, and country.

|  | Non-*H*30 | *H*30R1 | *H*30Rx | Total isolates | P value^b,c^ |
| --- | --- | --- | --- | --- | --- |
| Total | 13 (2.5) | 10 (1) | 12 (3) | 13 (3) | **< 0.001** |
| Spain | 10.5 (4) | 10 (0) | 12 (4) | 12 (3.75) | **0.001** |
| USA | 13 (1) | 10 (1) | 11.5 (1) | 11 (3) | **0.002** |
| P value^a,c^ | 0.79 | 0.79 | 0.1 | 0.1 |  |
| Pre-2014 | 12.5 (2) | 10 (1) | 11.5 (1) | 11 (2) | 0.01 |
| 2014 | 13 (3.25) | 10 (0) | 12 (4) | 12 (3.5) | **0.001** |
| P value^a,c^ | 0.51 | 0.79 | 0.1 | 0.012 |  |

^a^P value for the comparison of data in the column above.

^b^P value for the comparison of the data in the row.

^c^Chi-square and Fisher's exact tests were used for p values. After Bonferroni correction, only those p values < 0.008 were considered significant. Statistically significant values are in bold.

Table S6. Prevalence of ExPEC status in clonal subsets of the 84 *Escherichia coli* ST131 studied isolates by year of testing and country.

|  | Isolates (col %) tested pre-2014 | | | | Isolates (col %) tested in 2014 | | | |
| --- | --- | --- | --- | --- | --- | --- | --- | --- |
|  | Non-*H*30  n = 10 | *H*30R1 n = 13 | *H*30Rx n = 6 | Total^a^  n = 29 | Non-*H*30 n = 14 | *H*30R1 n =10 | *H*30Rx  n = 31 | Total^b^  n = 55 |
| ExPEC | 9 (90) | 5 (38) | 6 (100) | 20 (69) | 11 (79) | 4 (40) | 31 (100) | 46 (84) |
| Non-ExPEC | 1 (10) | 8 (62) | 0 (0) | 9 (31) | 3 (21) | 6 (60) | 0 (0) | 9 (16) |
|  | USA | | | | Spain | | | |
|  | Non-*H*30  n = 17 | *H*30R1 n = 13 | *H*30Rx n = 6 | Total  n = 36^c^ | Non-*H*30  n = 7 | *H*30R1 n = 10 | *H*30Rx  n = 31 | Total  n = 48d |
| ExPEC | 15 (88) | 5 (38) | 6 (100) | 26 (72) | 5 (71) | 4 (40) | 31 (100) | 40 (83) |
| Non-ExPEC | 2 (12) | 8 (62) | 0 (0) | 10 (28) | 2 (29) | 6 (60) | 0 (0) | 8 (17) |

Abbreviations: ExPEC, extraintestinal pathogenic *E. coli.*

^a^p = 0.003 for ExPEC status across clonal subsets for isolates tested pre-2014.

^b^p < 0.001 for ExPEC status across clonal subsets for isolates tested in 2014.

^c^p = 0.001 for ExPEC status across clonal subsets for isolates from USA.

^d^p < 0.001 for ExPEC status across clonal subsets for isolates from Spain.

Table S7. VG score of the different clonal subsets of the 84 *Escherichia coli* ST131 study isolates by ExPEC status, stratified by country or year.

| Year of testing/ Country of origin | | ExPEC status | VG score (median, IQR) of ST131 isolates by clonal subset | | | |
| --- | --- | --- | --- | --- | --- | --- |
|  |  |  | Non-*H*30 | *H*30R1 | *H*30Rx | Total |
| Year of testing | Pre-2014 | Non-ExPEC | 9 (0) | 10 (1) | - | 10 (1) |
|  |  | ExPEC | 13 (1) | 11 (0) | 11.5 (1) | 12 (2) |
|  |  | P value^a^ | 0.09 | **0.006** | NA^b^ | **< 0.001** |
|  | 2014 | Non-ExPEC | 10 (2) | 10 (1) | - | 10 (1) |
|  |  | ExPEC | 13 (2) | 10.5 (2.5) | 12 (4) | 12.6 (3) |
|  |  | P value^a^ | 0.009 | 0.055 | NA^b^ | **< 0.001** |
| Country | USA | Non-ExPEC | 8.5 (1) | 10 (1) | - | 9.5 (1) |
|  |  | ExPEC | 13 (1) | 11 (0) | 11.5 (1) | 12 (2) |
|  |  | P value^a^ | 0.02 | **0.006** | NA^b^ | **< 0.001** |
|  | Spain | Non-ExPEC | 10 (0) | 10 (1) | - | 10 (0.5) |
|  |  | ExPEC | 13 (3.5) | 10.5 (2.5) | 12 (4) | 12 (3.5) |
|  |  | P value^a^ | 0.05 | 0.06 | NA^b^ | **< 0.001** |
| Total | | Non-ExPEC | 9.5 (1.5) | 10 (1) | - | 10 (1) |
|  |  | ExPEC | 13 (1) | 11 (1) | 12 (3) | 12 (2) |
|  |  | P value^a^ | **0.002** | **0.001** | NA^b^ | **< 0.001** |

Abbreviations: ExPEC, extraintestinal pathogenic *E. coli.* VG score: virulence gene score.

^a^Chi-square and Fisher's exact tests were used for p values. After Bonferroni correction, only those p values < 0.008 were considered significant. Statistically significant values are in bold.

^b^NA: Not applicable. All *H*30Rx were ExPEC, so no VG score value was shown for non-ExPEC *H*30Rx isolates.

Table S8. Experimental virulence variables (ISS and “killer” status) of the 84 *Escherichia coli* ST131 studied isolates by clonal subset, stratified by year of testing or by country.

|  | Pre-2014 | | | | | 2014 | | | | |
| --- | --- | --- | --- | --- | --- | --- | --- | --- | --- | --- |
|  | Non-*H*30  n = 10 | *H*30R1  n = 13 | *H*30Rx  n = 6 | Total  n = 29 | P values^a^ | Non-*H*30 n = 14 | *H*30R1  n =10 | *H*30Rx  n = 31 | Total  n = 55 | P values^a^ |
| ISS (median, IQR) | 3.6 (1.9) | 2.3 (2.9) | 2.2 (1.5)^b^ | 2.5 (2.5) | 0.38 | 4.1 (1.7) | 2.8 (1.9) | 4.3 (1.5)^b^ | 4.3 (1.8) | 0.11 |
| Killer status (col %) | 5 (50) | 5 (38) | 0 (0)^c^ | 10 (34) | 0.11 | 8 (57) | 3 (30) | 23 (74)^c^ | 34 (62) | 0.03 |
|  | USA | | | | | Spain | | | | |
|  | Non-*H*30  n = 17 | *H*30R1  n = 13 | *H*30Rx  n = 6 | Total  n = 36 | P values^a^ | Non-*H*30  n = 7 | *H*30R1  n = 10 | *H*30Rx  n = 31 | Total  n = 48 | P values^a^ |
| ISS (median, IQR) | 3.8 (1.6) | 2.3 (2.9) | 2.2 (1.5)^d^ | 3.1 (2.4) | 0.13 | 4.4 (2.4) | 2.8 (1.9) | 4.3 (1.5)^d^ | 4.3 (1.9) | 0.12 |
| Killer status (col %) | 8 (47) | 5 (38) | 0 (0)^e^ | 13 (36) | 0.12 | 5 (71) | 3 (30) | 23 (74)^e^ | 31 (65) | 0.05 |

Abbreviations: ISS: Illness severity score.

^a^Chi-square and Fisher's exact tests were used for p values. After Bonferroni correction, only those p values < 0.008 were considered significant.

^b^P value for the comparison of ISS in *H*30Rx isolates across years: p = 0.004.

^c^P value for the comparison of “killer” fraction in *H*30Rx isolates across years: p = 0.001.

^d^P value for the comparison of ISS in *H*30Rx isolates across countries: p = 0.004.

^e^P value for the comparison of “killer” fraction in *H*30Rx isolates across countries: p = 0.001.

Table S9. Experimental virulence variables (ISS and “killer” status) of the 84 *Escherichia coli* ST131 studied isolates by ExPEC status, stratified by year of testing or by country.

| Year/Country | ExPEC status | ISS  (median, IQR) | | P value^a^ | Killer status (row %) | P value^a^ |
| --- | --- | --- | --- | --- | --- | --- |
| Pre-2014 | ExPEC (n = 20) | 3.6 (2.3) | **0.003** | | 45 % | 0.11 |
|  | Non-ExPEC (n = 9) | 1.9 (1.0) |  |  | 11 % |  |
| 2014 | ExPEC (n = 46) | 4.3 (1.7) | **0.03** | | 70 % | **0.02** |
|  | Non-ExPEC (n = 9) | 2.9 (2.1) |  |  | 16 % |  |
| USA | ExPEC (n = 26) | 3.8 (2.2) | **0.002** | | 46 % | 0.06 |
|  | Non-ExPEC (n = 10) | 2.0 (1.0) |  |  | 10 % |  |
| Spain | ExPEC (n = 40) | 4.3 (1.6) | **0.04** | | 72 % | **0.02** |
|  | Non-ExPEC (n = 8) | 2.7 (2.5) |  |  | 25 % |  |

Abbreviations: ExPEC, extraintestinal pathogenic *E. coli.* ISS: Illness severity score

^a^Chi-square and Fisher's exact tests were used for p values. After Bonferroni correction, only those p values ≤ 0.02 were considered significant. Significant values are in bold.
